# Breathing exercises improve post-operative pulmonary function and quality of life in patients with lung cancer: A meta-analysis

**DOI:** 10.3892/etm.2013.926

**Published:** 2013-01-25

**Authors:** WEI LIU, YING-LI PAN, CAI-XIANG GAO, ZUO SHANG, LI-JUAN NING, XING LIU

**Affiliations:** 1 Departments of General Surgery, The Fourth Affiliated Hospital of China Medical University, Shenyang, Liaoning 110032, P.R. China; 2 Nursing, The Fourth Affiliated Hospital of China Medical University, Shenyang, Liaoning 110032, P.R. China; 3 Cardiology, The Fourth Affiliated Hospital of China Medical University, Shenyang, Liaoning 110032, P.R. China

**Keywords:** breathing exercises, lung cancer, pulmonary function, quality of life, meta-analysis

## Abstract

Previous research has shown that breathing exercises may improve the prognosis and health status in patients with lung cancer by enhancing pulmonary function and quality of life (QOL). However, individually published results are inconclusive. The aim of the present meta-analysis was to evaluate the clinical value of breathing exercises on post-operative pulmonary function and QOL in patients with lung cancer. A literature search of Pubmed, Embase, the Web of Science and CBM databases was conducted from their inception through to October 2012. Crude standardized mean differences (SMDs) with 95% confidence intervals (CIs) were used to assess the effect of breathing exercises. A total of eight clinical studies were ultimately included with 398 lung cancer patients. When all the eligible studies were pooled into the meta-analysis, there was a significant difference between the pre-intervention and post-intervention results of breathing exercises on post-operative pulmonary function; forced expiratory volume in 1 sec (FEV1): SMD, 3.37; 95% CI, 1.97–4.77; P<0.001; FEV1/FVC: SMD, 1.77; 95% CI, 0.15–3.39; P=0.032). Furthermore, the QOL in patients with lung cancer was significantly improved following the intervention with breathing exercises; there were significant differences between the pre-intervention and post-intervention results on the ability of self-care in daily life (SMD, −1.00; 95% CI, −1.467 to −0.52; P<0.001), social activities (SMD, −0.94; 95% CI, −1.73 to −0.15; P=0.02), symptoms of depression (SMD, −0.91; 95% CI, −1.25 to −0.57; P<0.001) and symptoms of anxiety (SMD, −0.91; 95% CI, −1.20 to −0.63; P<0.001). Results from the present meta-analysis suggest that breathing exercises may significantly improve post-operative pulmonary function and QOL in patients with lung cancer.

## Introduction

Lung cancer is the most common cause of cancer mortality in males and females worldwide (1). The World Health Organization estimates that worldwide lung cancer mortality should continue to rise, largely due to the increase in global tobacco smoke, which is the main risk factor responsible for 80–90% of all lung carcinomas (2). Non-smokers account for only 10–15% of the incidence of lung cancer, which is often attributed to a combination of genetic factors, occupational exposure, including radon gas and asbestos, air pollution and second hand smoke (3). Clinical therapies of lung cancer are principally composed of surgery, radiation, chemotherapy, targeted therapy and palliative care, alone or in combination, in an attempt to cure or lessen the adverse impact of malignant neoplasms originating in the lung tissue (4,5). Surgical resection remains the first choice of therapy for the majority of patients with lung cancer (6). However, all curative therapies for lung cancer inevitably result in certain negative effects with regard to post-operative pulmonary function and quality of life (QOL) following rehabilitation (7). Breathlessness, alongside coughing, for instance, is the most common depressing secondary symptom in lung cancer patients, which may lead to physical disability, loss of independence and dignity and lowered self-esteem with associated psychosocial distress, thereby severely affecting the QOL of patients with lung cancer (8). Moreover, breathlessness is a poorly controlled symptom against which traditional pharmacological interventions, including benzodiazepines, opioids and oxygen, are often ineffective (9). Therefore, breathing exercises as a non-pharmacological approach to improve post-operative pulmonary function and QOL in lung cancer patients have attracted increasing attention in recent years.

Breathing exercises aim to correct breathing errors, reestablish a proper breathing pattern, increase diaphragm activity, elevate the amount of alveolar ventilation, reduce energy consumption when breathing and relieve the shortness of breath experienced by patients with lung cancer. Several studies have shown that a number of patients with lung cancer fear the possibility of suffocation when they feel breathless during physical activities such as climbing stairs (10,11). In order to combat this shortness of breath, breathinxg exercises are used as an alternative treatment to bolster post-operative pulmonary function by teaching patients to utilize more of their lungs (12). In their simplest form, breathing exercises consist of elongating and slowing down the inhalation and exhalation, which allow lung cancer patients to take deeper breaths that increase their intake of oxygen, rather than taking shallow breaths that only make use of the top half of their lungs (13). Previously, studies examining the effect of breathing exercises on improvements to post-operative pulmonary function and QOL following rehabilitation showed inconclusive results. However, the majority of studies came to the conclusion that lung cancer patients suffering from breathlessness benefited from breathing exercises with regard to the aspects of post-operative pulmonary function and QOL (14–16). Certain other studies have not demonstrated the favorable effects of breathing exercises on post-operative pulmonary function and QOL in patients with lung cancer (17). Given these circumstances, a meta-analysis was performed to evaluate the clinical value of breathing exercises on post-operative pulmonary function and QOL in patients with lung cancer.

## Materials and methods

### Literary search strategy

Relevant manuscripts published prior to October 1st 2012 were identified through a search of Pubmed, Embase, the Web of Science and CBM databases using the following terms: ‘lung neoplasms’, ‘pulmonary neoplasms’, ‘pulmonary neoplasm’, ‘lung cancer’ or ‘bronchial neoplasms’; and ‘breathing exercises’, ‘exercise of breathing’, ‘respiratory muscle training’ or ‘training of respiratory muscle’. Eligible articles or textbooks were also reviewed and checked via manual searches to find other potential studies. Any disagreements were resolved by discussion between the authors.

### Inclusion and exclusion criteria

To be eligible for inclusion in the present meta-analysis, the following criteria were established: i) The study must be a clinical study focused on the effect of breathing exercises on post-operative pulmonary function and QOL in patients with lung cancer; ii) all patients diagnosed with lung cancer should have had confirmation from a pathological examination of the surgical specimen; iii) the patients in the treatment group must have been assigned to receive breathing exercises, including inspiratory muscle training, simple relaxation techniques, activity pacing or psychosocial support, under the guidance of their physicians and nurse; and iv) sufficient data must be published on the outcomes of the breathing exercises, including pulmonary function, QOL, visual analog scales (VAS), hospital anxiety and depression. Studies were excluded when they were: i) Not clinical studies that evaluated the clinical value of breathing exercises on post-operative pulmonary function and QOL in patients with lung cancer; ii) case reports, letters, reviews, meta-analyses and editorial articles; iii) studies that were based on incomplete raw data and those with no usable data reported; and iv) duplicates of previous publications.

### Data extraction

Using a standardized form, data from the published studies were extracted independently by two reviewers to populate the necessary information. For each study, the following characteristics were collected: the first author, year of publication, country, language, study design, number of cases, ethnicity, inclusion criteria, pathological type, follow-up period and outcome index. In cases of conflicting analysis, an agreement was reached following a discussion between the authors. If required, a third reviewer confirmed any discrepancies or uncertainties related to the data abstraction process.

### Quality assessment of the included studies

The methodological quality of the included studies, including randomization, similarity of groups, co-intervention, masking, outcome measures, compliance, exercise regime and follow-up, was evaluated by two independent reviewers using a modified methodological quality scale (18). A total of eight assessment items matching with the quality appraisal were used in this meta-analysis with scores ranging from 0 to 40 (5 scores for each item). Scores of 0–19, 20–29 and 30–40 were defined as low, moderate and high quality, respectively. The two reviewers resolved any differences of opinion by discussion.

### Statistical analysis

The differences between the pre-intervention and post-intervention results of breathing exercises on post-operative pulmonary function and QOL were measured by standardized mean differences (SMDs) with 95% confidence intervals (CIs). The statistical significance of the pooled SMD was examined by Z-test. Between-study variations and heterogeneities were estimated using Cochran’s Q statistic and P<0.05 was considered to indicate a statistically significant heterogeneity (19). The effect of heterogeneity was also quantified using the I^2^ test, which ranges from 0–100% and represents the proportion of inter-study variability that may be contributed by heterogeneity rather than by chance (20). When the Q-test was significant (P<0.05) or I^2^>50% this indicated that heterogeneity existed among the studies and the random-effects model (DerSimonian-Laird method) was conducted in the meta-analysis. Otherwise, the fixed-effects model (Mantel-Haenszel method) was used. A sensitivity analysis was performed by omitting each study in turn to assess the stability of the results. Begg’s funnel plots and Egger’s linear regression tests, which measure funnel plot asymmetry, were used to detect any publication bias (21). All the P-values were two-sided and P<0.05 was considered to indicate a statistically significant difference. All analyses were calculated using STATA Version 12.0 software (Stata Corp, College Station, TX, USA).

## Results

### Characteristics of the included studies

**Figure 1:**
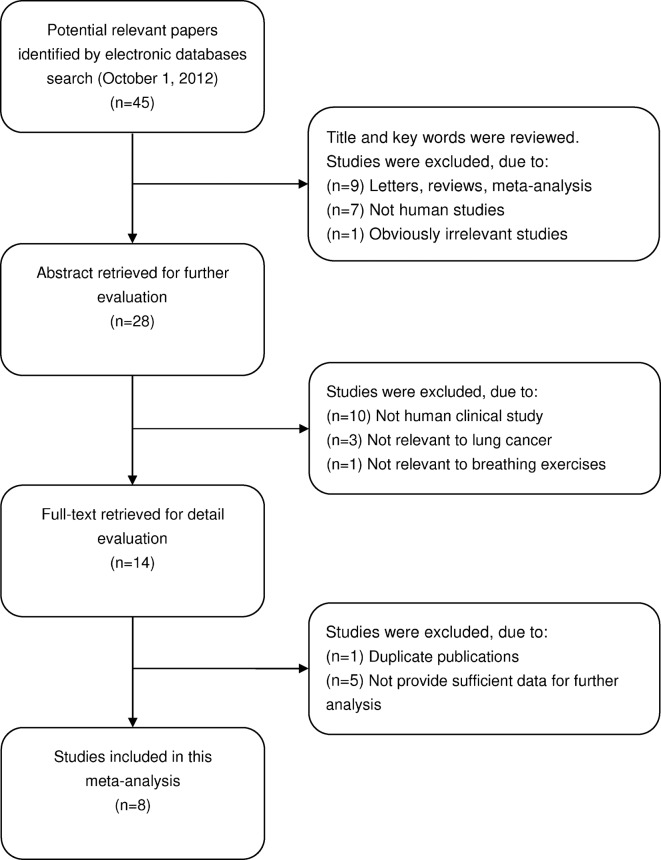
Flow chart of the literature search and study selection process.

In total, 45 potentially relevant studies were identified by searching electronic databases. According to the inclusion criteria, 8 clinical studies (8,15–17,22–25) were included and 37 articles were excluded in the present meta-analysis. The details of the selection process are presented in a flow chart in Fig. 1. The publication year for the involved studies ranged from 1996 to 2012. A total of 398 lung cancer patients were included in these eight studies, which all evaluated the effect of breathing exercises on post-operative pulmonary function and QOL in patients with lung cancer. All patients fulfilled the diagnostic criteria of lung cancer, as confirmed by pathological examination of surgical specimens. Five studies had been carried out in China and three studies in the UK. According to the modified methodological quality scale, the scores of all the included studies were moderately high (>20 points) and varied from 20 to 31 (median, 24). The main characteristics and methodological quality of all the eligible studies are listed in Table I. A summary of the differences between the pre-intervention and post-intervention results for post-operative pulmonary function and QOL is provided in Table II.

### Pulmonary function

**Figure 2:**
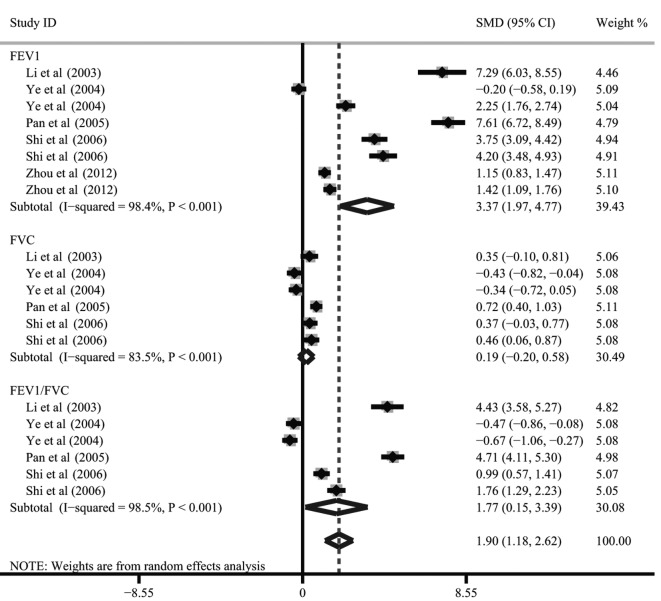
Forest plot of ORs with a random-effects model for the difference between pre-intervention and post-intervention results of breathing exercises for pulmonary function. OR, odds ratio; SMD, standardized mean difference; CI, confidence interval; FEV1, forced expiratory volume in 1 sec; FVC, forced vital capacity.

The difference between the pre-intervention and post-intervention results of breathing exercises on pulmonary function was investigated in five studies. There were three main outcome indices, including the forced vital capacity (FVC), the forced expiratory volume in 1 sec (FEV1) and the the ratio of FEV1/FVC. The heterogeneity was significant (all P<0.05) so the random-effects model was used. When all five studies were pooled into the meta-analysis, there was a significant difference between the pre-intervention and post-intervention results of breathing exercises on FEV1 and FEV1/FVC (FEV1: SMD, 3.37; 95% CI, 1.97–4.77; P<0.001; FEV1/FVC: SMD, 1.77; 95% CI, 0.15–3.39; P=0.032), but no difference was identified for FVC (SMD, 0.19; 95% CI, −0.20–0.58; P=0.336; Fig. 2).

### QOL

**Figure 3:**
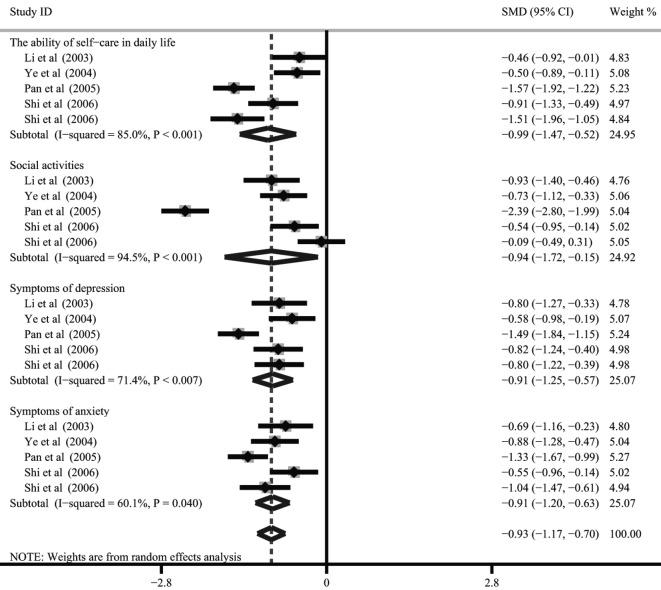
Forest plot of ORs with a random-effects model for the differences between the pre-intervention and post-intervention results of breathing exercises on quality of life. OR, odds ratio; SMD, standardized mean difference; CI, confidence interval.

There were four studies [Li (24), Ye *et al* (17), Pan *et al* (15) and Shi (16)] that referred to the differences between the pre-intervention and post-intervention results of breathing exercises on QOL. The four main outcome indices that were addressed were the ability of self-care in daily life, engagement in social activities, the symptoms of depression and the symptoms of anxiety. Since heterogeneity existed (all P<0.05), the random-effects model was conducted to pool the results. The meta-analysis results indicated that the QOL in patients with lung cancer was significantly improved following the intervention with breathing exercises. There were significant differences between the pre-intervention and post-intervention results of breathing exercises on the ability of self-care in daily life (SMD, −0.99; 95% CI, −1.47 to −0.52; P<0.001), engagement in social activities (SMD, −0.936; 95% CI, −1.725 to −0.148; P=0.02), the symptoms of depression (SMD, −0.91; 95% CI, −1.25 to −0.57; P<0.001) and the symptoms of anxiety (SMD, −0.91; 95% CI, −1.20 to −0.63; P<0.001; Fig. 3).

### VAS

**Figure 4:**
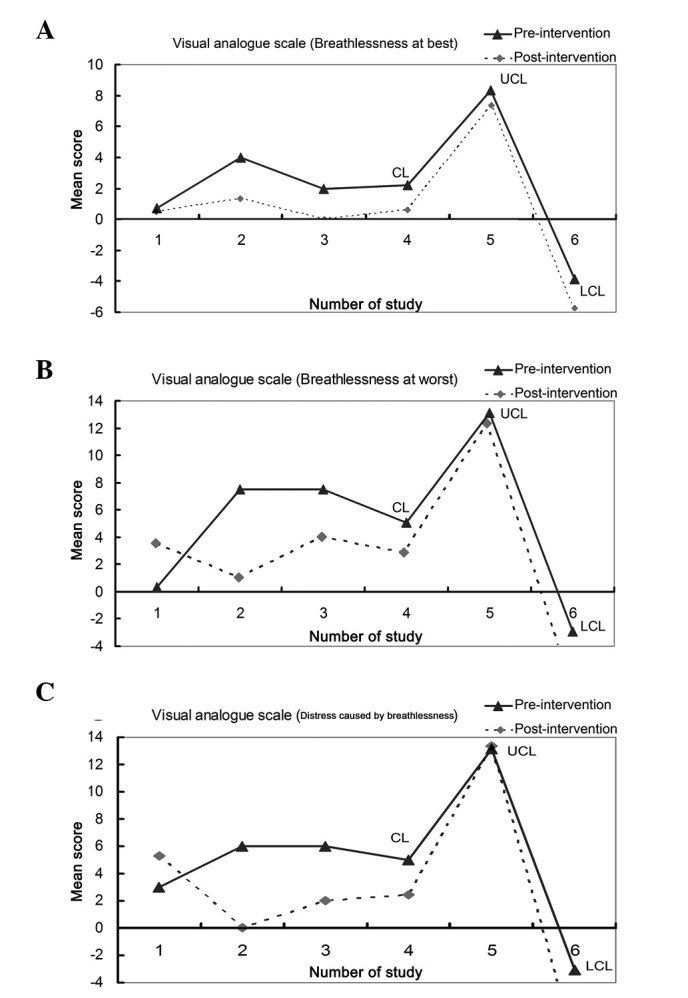
Broken-line graphs for the difference between the pre-intervention and post-intervention results of breathing exercises on visual analog scales: (A) Breathlessness at worst; (B) breathlessness at best; and (C) distress caused by breathlessness. UCL, upper control limit; LCL, lower control limit; CL, control limit.

Only three studies referred to the differences between the pre-intervention and post-intervention results of breathing exercises in the VAS (8,22,23). The three main outcome indices addressed were breathlessness at worst, breathlessness at best and distress caused by breathlessness. Due to limited data, the analysis of this was only qualitative. No significant differences were observed between the pre-intervention and post-intervention results of breathing exercises for the VAS in the three broken-line graphs (Fig. 4).

### Hospital anxiety and depression

**Figure 5:**
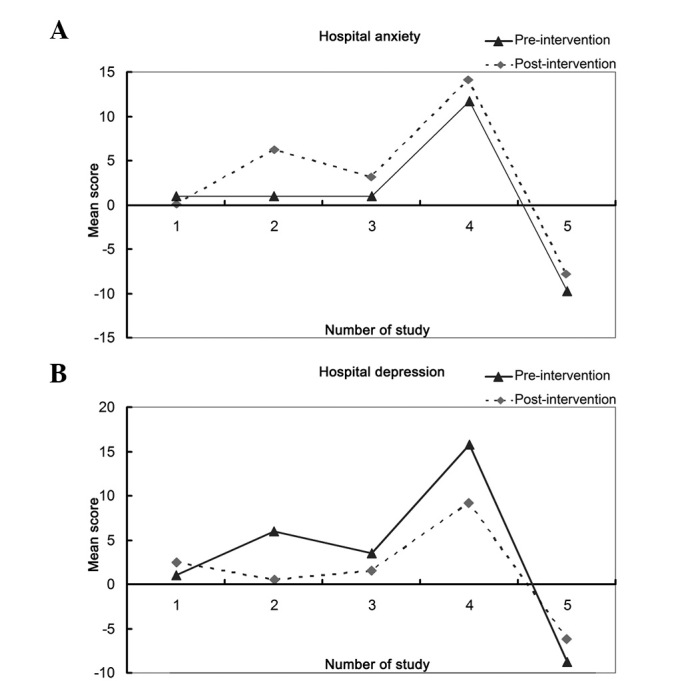
Broken-line graphs for the differences between the pre-intervention and post-intervention results of breathing exercises on (A) hospital anxiety; and (B) hospital depression. UCL, upper control limit; LCL, lower control limit; CL, control limit.

There were also only two studies (22,23) that referred to the differences between the pre-intervention and post-intervention results of breathing exercises on hospital anxiety and depression. Qualitative data analysis showed that there were no significant differences between these results (Fig. 5).

### Sensitivity analysis and publication bias

**Figure 6:**
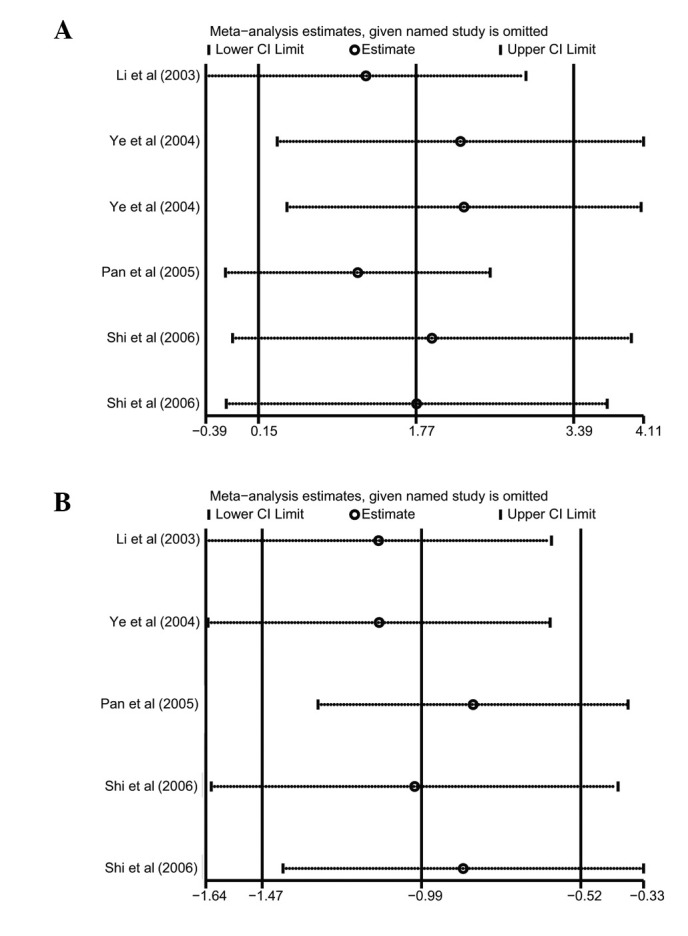
Sensitivity analysis of pulmonary function and QOL analysis. Results were computed by omitting each study in turn. Meta-analysis random-effects estimates were used. The two ends of the dotted lines represent the 95% CI. QOL, quality of life; CI, confidence interval.

A sensitivity analysis was performed to assess the effect of each individual study on the pooled SMD of pulmonary function and QOL analysis by omission of individual studies. The analysis results suggested that no individual study significantly affected the pooled values of the clinical events (Fig. 6), indicating that the results of the present study are statistically robust.

**Figure 7:**
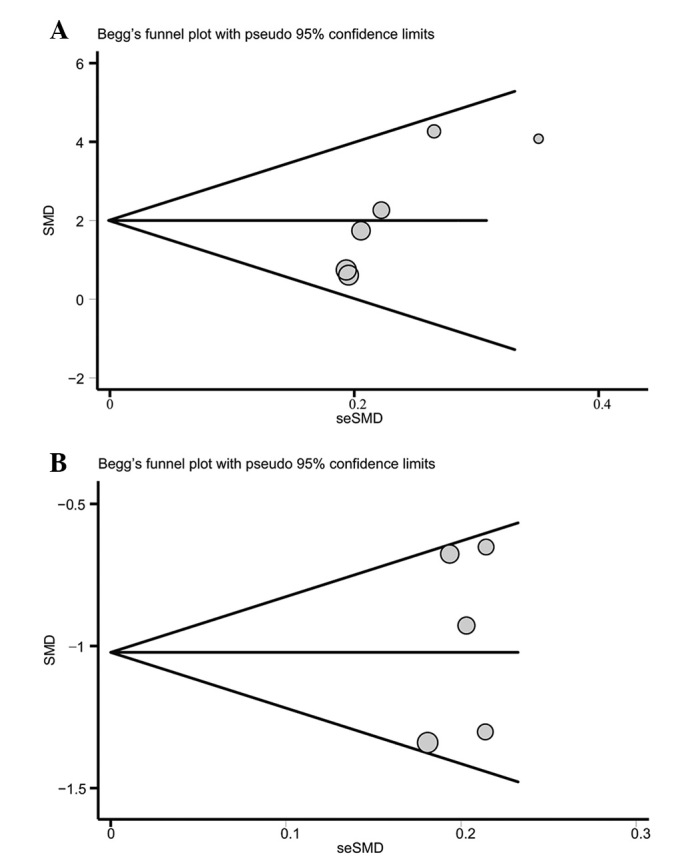
Begg’s funnel plot of publication bias in pulmonary function and quality of life analysis. SMD, standardized mean difference; seSMD, standard error of standardized mean difference.

Publication bias exists to the extent that available research results are unrepresentative of all research results. A Begg’s funnel plot and Egger’s linear regression test were performed to assess the publication bias of the included studies. The shapes of the funnel plots for pulmonary function and QOL analysis did not reveal any evidence of marked asymmetry (Fig. 7). Egger’s test also showed that there was no statistically significant evidence of a publication bias (pulmonary function: t=2.47, P=0.062; QOL: t=0.70, P=0.534).

## Discussion

Currently, surgical therapy for lung cancer is aimed not only at prolonging survival periods, but additionally at improving post-operative QOL, which is also the ultimate goal of effective cancer treatment (5). However, due to the impact of multiple factors subsequent to surgery, including anesthesia, wound pain, pleural reaction and pleural adhesions, there is an inevitable decline in respiratory function, breathing difficulty, abnormal lung capacity ventilation, reduced effective diffusion area and an imbalanced ventilation/perfusion ratio, to various degrees (6). In order to correct these aberrant pulmonary functions, the body spontaneously over-utilizes auxiliary respiratory muscles and thereby forms an improper breathing pattern. The formation of an incorrect pattern of breathing not only fails to relieve irregular post-operative symptoms, but also makes patients with lung cancer more susceptible to respiratory muscle fatigue, hypoxia and carbon dioxide retention, which may eventually cause chronic obstructive pulmonary disease and respiratory failure, including breathlessness (13). The occurrence of breathlessness may seriously affect the length of survival, self-care ability, labor and interpersonal skills of patients with lung cancer and accordingly decrease QOL and lead to psychological depression and anxiety (9).

Breathing exercises have long been recognized as an effective method to reduce the post-operative complications of lung cancer, including breathlessness, and thus improve pulmonary function and QOL by strengthening the respiratory muscles (16). Breathing exercises may be categorized into specific and non-specific respiratory muscle training. Specific breathing exercises, including lip reduction and deep abdominal breathing exercises, are conducted primarily in a pressurized respiratory manner. Generally, lip reduction breathing exercises refer to the nasal inspiratory and lip reduction expiratory breathing patterns caused by shrinking the lips, as if whistling, to slowly exhale the gas and then maintaining this for >10 sec (14). Deep abdominal breathing exercises allow patients to train in a sitting, supine or lateral position, and requires concentration, natural postures, relaxation of the muscles and a gradual deepening of breathing to reach a maximum lung capacity. The air is then excluded for 10 sec and the patient should exhale slowly (14). In addition, non-specific breathing exercises are often identified as whole body exercises, including stair climbing, qigong, breathing gymnastics and balloon blowing (26). The applications of proper breathing exercises, together with symptomatic care and a comprehensive and timely assessment of the physical and psychological state of patients with lung cancer, show promise in relieving the symptoms of breathlessness and in improving the post-operative pulmonary function and QOL following rehabilitation (27).

However, studies investigating the improvements to post-operative pulmonary function and QOL caused by breathing exercises have suggested conflicting results. Therefore, the present meta-analysis of all eligible studies was performed to evaluate the exact effects of breathing exercises on post-operative pulmonary function and QOL in patients with lung cancer. A significant enhancement in pulmonary function was observed in FEV1 and FEV1/FVC following the use of breathing exercises, but no significant differences were identified in FVC between the pre-intervention and post-intervention results of breathing exercises. These results were inconsistent with those of several previous studies (14–16). This may have been due to the deviation of the instruments measuring the pulmonary function parameters, the baseline characteristics (such as histological type, differentiation and disease stage) of the patients or the duration of the follow-up. Additionally, the present study demonstrated a significant reduction in the symptoms of depression and anxiety caused by breathlessness and a decrease in the promotion of the ability to perform self-care for daily living and to engage in social activities, indicating an improvement in the post-operative QOL of the patients with lung cancer. The outcomes of the VAS, which are usually utilized to measure the degree of breathlessness, were also analyzed and no significant differences were observed in the degree of breathlessness between the pre-intervention and post-intervention results of the breathing exercises.

In interpreting the results of the current meta-analysis, specific issues pertinent to this study need to be addressed. Firstly, the sample size included in the present study is relatively small and may have overestimated the clinical values of breathing exercises on post-operative pulmonary function and QOL in patients with lung cancer. In addition, the origins of heterogeneity may include a number of factors, including criteria, characteristics of the patients and the follow-up period. A selection bias may exist due to the differences in the mean age of the subjects, the duration of the intervention, the inclusion criteria or the study design. Finally, although all the participants in each study were well defined with similar inclusion criteria, there may be potential factors that have not been taken into account that may have affected the results and they should therefore be interpreted with caution owing to the potential heterogeneity among trials.

In conclusion, this meta-analysis provides strong evidence that breathing exercises may significantly improve post-operative pulmonary function and QOL in patients with lung cancer. Based on the limitations mentioned, larger clinical trials are required to confirm these findings. Further studies investigating the role of breathing exercises following surgical therapy are also required.

## Figures and Tables

**Table I:** Characteristics of studies included in the present meta-analysis.

First author (ref.)	Year	Country	Ethnicity	Study design	Case number	Duration of intervention	Frequency of sessions	Main outcome measures	Quality scores
Corner *et al* (22)	1996	UK	Caucasian	RCT	11	3–6 weeks	Weekly	Visual analog scales, hospital anxiety and depression scale	30
Bredin *et al* (23)	1999	UK	Caucasian	RCT	51	3–8 weeks	Weekly	Visual analog scales, hospital anxiety and depression scale	26
Hately *et al* (8)	2003	UK	Caucasian	Cohort study	30	4–6 weeks	Weekly	Visual analog scales	25
Li (24)	2003	China	Asian	Cohort study	38	6 months	6 months	Pulmonary function, quality of life	21
Ye *et al* (17)	2004	China	Asian	Cohort study	52	6 months	3 months	Pulmonary function, quality of life	22
Pan *et al* (15)	2005	China	Asian	Cohort study	82	1–6 months	6 months	Pulmonary function, quality of life	20
Shi (16)	2006	China	Asian	Cohort study	48	6 months	3 months	Pulmonary function, quality of life	23
Zhou *et al* (25)	2012	China	Asian	Cohort study	86	1 week	1 week	Pulmonary function	22

RCT, randomized controlled trial.

**Table II:** Summary of the effect of breathing exercises on post-operative pulmonary function and quality of life.

Parameters	SMD	95% CI	P-value	P_h_	I^2^ (%)
Pulmonary function					
FEV1	3.369	1.968, 4.770	<0.001^a^	<0.001	98.40
FVC	0.192	−0.199, 0.582	0.336^a^	<0.001	83.50
FEV1/FVC	1.77	0.148, 3.392	0.032^a^	<0.001	98.50
Quality of life					
The ability of self-care in daily life	−0.992	−1.467, −0.517	<0.001^a^	<0.001	85.00
Social activities	−0.936	−1.725, −0.148	0.02^a^	<0.001	94.50
Symptoms of depression	−0.911	−1.249, −0.572	<0.001^a^	0.007	71.40
Symptoms of anxiety	−0.914	−1.201, −0.628	<0.001^a^	0.04	60.10

SMD, standardized mean difference; CI, confidence interval; P_h_, P-value of heterogeneity test; FEV1, forced expiratory volume in 1 sec; FVC, forced vital capacity;

a Estimates for random-effects model.
